# Bilateral Absence of the Zygomatic Nerve and Zygomaticofacial Nerve and Foramina

**DOI:** 10.7759/cureus.1505

**Published:** 2017-07-23

**Authors:** Shehzad Khalid, Joe Iwanaga, Marios Loukas, R. Shane Tubbs

**Affiliations:** 1 Department of Anatomical Sciences, St. George's University School of Medicine, Grenada, West Indies; 2 Seattle Science Foundation; 3 Neurosurgery, Seattle Science Foundation

**Keywords:** zygoma, foramen, nerve, anatomy, cadaver, variations

## Abstract

The zygomaticofacial branch (ZFb) of the zygomatic nerve travels along the inferolateral angle of the orbit, traverses the zygomaticofacial foramen (ZFF) in the zygomatic bone, and then perforates the orbicularis oculi muscle to finally reach the skin of the malar area, which it innervates. The bilateral absence of the ZFb and the ZFF was found in an 80-year-old Caucasian cadaver. In addition, both zygomatic nerves were absent. A thin nerve arising from the lacrimal nerve passed below it and gave rise to the lacrimal branch and a communicating branch to the lacrimal nerve. This then entered the small bony canal, which opened at the medial aspect of the lateral wall of the orbit on the right and left sides. The bilateral absence of the ZFb of the zygomatic nerve and its foramen appears to be uncommon but should be realized during surgery or invasive procedures over the cheek or infraorbital region. The additional absence of both zygomatic nerves is exceptional.

## Introduction

The malar surface of the zygomatic bone is convex and perforated near its center with a small aperture, the zygomaticofacial foramen (ZFF), where the zygomaticofacial branch (ZFb) and vessels traverse to reach the face. The ZFb perforates the orbicularis oculi muscle to reach the skin of the cheek. It joins with the zygomatic branches of the facial nerve and with the inferior palpebral branches of the maxillary nerve (V2) [1]. The zygomatic nerve and its branches can be easily injured during facial surgery in the periorbital region [2]. A postoperative hematoma may occur by tearing the vessels arising from the ZFF if blind surgical dissections were to be done in this area. Many cases have reported damage to the ZFb involving osteotomies in the malar area. It is vital that the zygomaticofacial vessels be identified and coagulated to prevent avulsion, bleeding, and complications related to postoperative hematomas since these vessels can be disrupted by elevating the periorbita from the lateral orbital wall [1]. The zygomatic bone shows variations in the number of the foramina on its facial aspect. Occasionally, the nerve and the foramen have been found to be absent [3]. We report a case of the bilateral absence of the ZFb and ZFF and evaluate the intraorbital anatomy of the zygomatic nerve in this case.

## Case presentation

**Figure 1:**
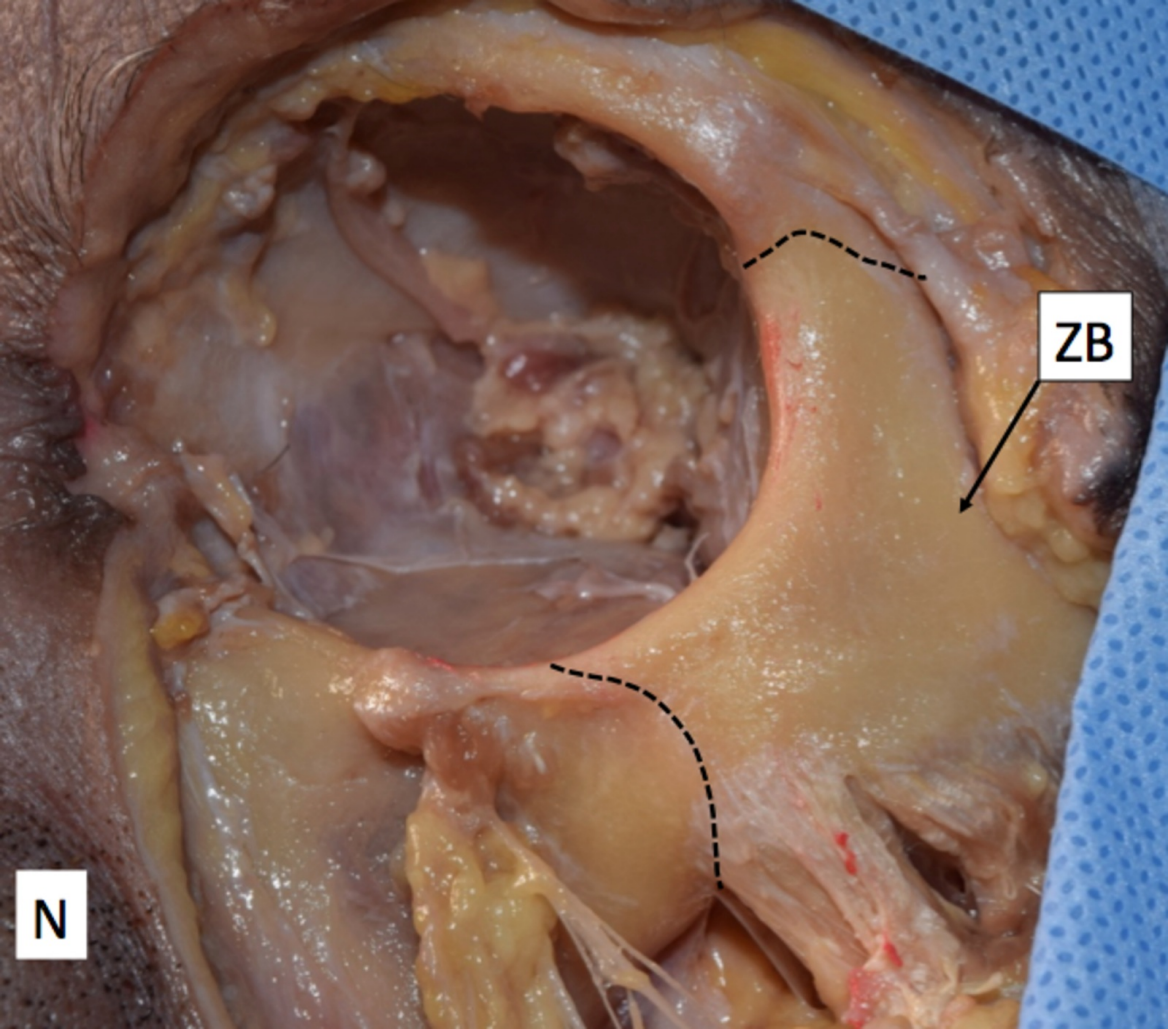
Surface of the zygomatic bone Note that no zygomaticofacial foramen is present. N: Nose; ZB: Zygomatic bone

During our routine dissection of the face of a fresh cadaver, a bilateral absence of the ZFb and ZFF was found in a Caucasian female whose age at death was 80 (Figure 1).

**Figure 2:**
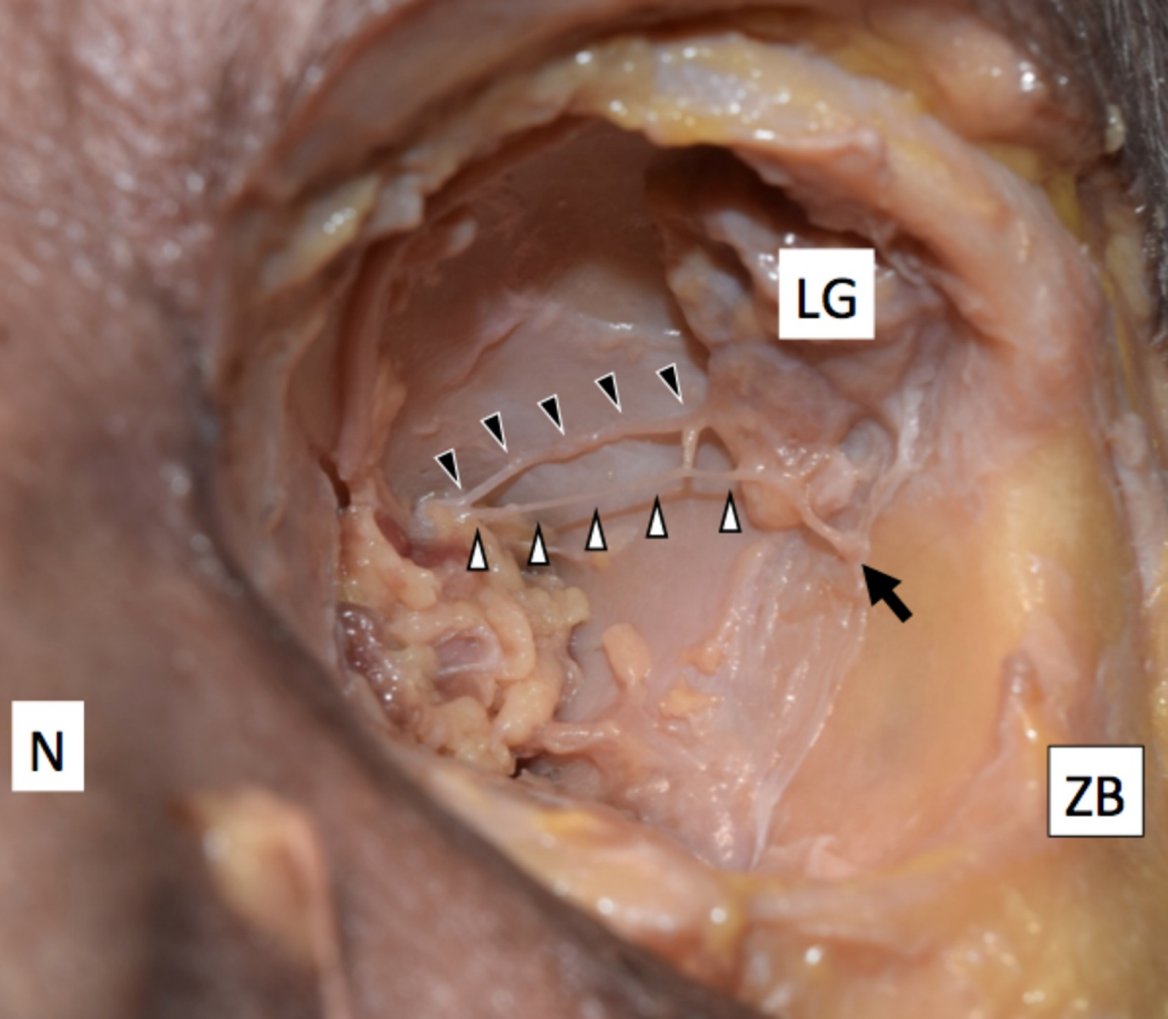
Zygomaticotemporal branch of the lacrimal nerve The zygomaticotemporal branch (white arrowheads) arising from the lacrimal nerve (black arrowheads) and entering a bony canal (arrow). LG: Lacrimal gland; N: Nose; ZB: Zygomatic bone

Inside the orbit, neither the zygomatic nerve nor the ZFb was seen. A thin nerve arose from the lacrimal nerve, passed below it, and gave rise to the lacrimal branch and a communicating branch with the lacrimal nerve. This nerve then entered a small bony canal, which opened into the medial aspect of the lateral wall of the orbit on both the right and left sides (Figure 2). The diameter of this nerve was 0.67 mm on the right and 0.62 mm on the left side, respectively. This nerve was considered the ZFb of the lacrimal nerve. 

Dissection was performed under a surgical microscope (OPMI CS NC31, Carl Zeiss, Oberkochen, Germany), and measurement was made with a microcaliper (Mitsutoyo, Kanagawa, Japan). The study was conducted in accordance with the requirements of the Declaration of Helsinki (64th WMA General Assembly, Fortaleza, Brazil, October 2013).

## Discussion

The prevalence of the ZFF has been reported by Aksu et al., where 80 adult dry skulls (160 sides) of West Anatolian people were studied [4]. They measured the distance from the ZFF to the midpoint of the frontozygomatic suture (ZFF-FZ), to the lowest point of the zygomaticomaxillary suture (ZFF-ZMS), and to the closest point of the orbital rim (ZFF-OK). When more than one foramen was found, the largest one was taken as the point of reference and the measurements described earlier were performed [4]. The mean ZFF-FZ was 26.2 ± 3.2 mm, the mean ZF-ZMS was 18.6 ± 3.1 mm, and the mean ZFF-OK was 5.9 ± 1.4 mm for both sides. There was no statistically significant difference between the right and left sides. The number of ZFFs on each side was as follows: one on 71 sides (44.4 percent), two on 45 sides (28.1 percent), three on 10 sides (6.3 percent), four on 7 sides (4.4 percent), and five on 2 sides (1.3 percent) [4]. There were no foramina on 25 sides (15.6 percent) [4]. Meanwhile, in a study by Mangal et al. (2004), there was no ZFF on 11.5 percent and 10.3 percent on the right and left sides, respectively. The ZFF has often been reported as doubled [1] or absent [5]. Occasionally, the ZFb, which travels through the ZFF does not exist [1], and in these cases, the inferior palpebral branches of the infraorbital nerve supply the area normally covered by the ZFb. A recent study by Iwanaga et al. [6] also found an additional inferior palpebral branch of the infraorbital nerve arising from the superior surface of the infraorbital canal, which has not been previously described. This could also explain why sensory innervation is still intact after surgical dissection with damage to this nerve [7].

Both the ZFb of the zygomatic nerve and the zygomatic nerve were absent in our case. The zygomaticotemporal branch (ZTb) usually arises from the zygomatic nerve, traverses a canal in the zygomatic bone, and emerges into the anterior part of the temporal fossa [1]. It occasionally has a communicating branch with the lacrimal nerve. In this case, the ZTb arose from the lacrimal nerve and had a communicating branch with the lacrimal nerve.

The zygomatic bone may have one to three ossification centers. These appear in the eighth week of fetal life and fuse in the twenty-second week [2]. The embryological origin of the variations of the ZFF may be due to the variations of its ossification centers. As the ossification of the bone and related foramina are induced following the formation of the enclosed nerve [8], an absence of the nerve could result in the absence of the foramen.

## Conclusions

The absence of both the ZFF and its ZFb appears to be uncommon. The additional bilateral absence of zygomatic nerves is exceptional. Procedures in adjacent areas of the infraorbital region should keep this in mind so as not to deinnervate the skin over the cheek by injuring a branch of the infraorbital nerve that would be present there.
